# Propofol suppresses hepatocellular carcinoma by inhibiting NET1 through downregulating ERK/VEGF signaling pathway

**DOI:** 10.1038/s41598-020-67693-0

**Published:** 2020-07-08

**Authors:** Guoxiong Fei, Meili Cao, Chunlin Ge, Longjiu Cui

**Affiliations:** 1 Department of Anaesthesiology, Shanghai Xuhui Central Hospital, Shanghai, 200031 China; 2 Department of Anaesthesiology, Eastern Hepatobiliary Surgical Hospital, Shanghai, 200438 China; 3 Division of Laparoscopic, Eastern Hepatobiliary Surgical Hospital, Shanghai, 200438 China

**Keywords:** Biogeochemistry, Genetic engineering

## Abstract

Hepatocellular carcinoma (HCC) is the leading cause of tumor death in China with high mortality since its strong metastatic potential. Currently, treatment against advanced HCC is poorly efficient and thus screening new drugs to prevent the HCC invasion is of great significance to improve the survival rate of patients with HCC. From the results of this study, we concluded that propofol, a widely used anesthetics could prevent the proliferation by MTT assay. The scratch wound and invasion assays showed that migratory property and invasiveness in HCC cells SMMC-7721 was inhibited by propofol. This process was probably mediated by NET1 since NET1 overexpression offset the repressive effect of propofol on the invasiveness and migratory ability of SMMC-7721 cells. Furthermore, propofol treatment also reduced p-ERK1/2 and VEGF level by western blot analysis. Similar observation was found when NET1 was silenced. Thus, the results of this study provided valuable clinical therapy potential of propofol against liver cancer. We also disclosed molecular mechanism underlying the regulation of invasion and migration in HCC cells by NET1.

## Introduction

Liver cancer is a fatal cancer and presented the second mortality rate in the world^1,2^. Hepatocellular carcinoma (HCC) belonging to primary liver cancer^3^ acts as the third leading mortality of tumor‑related deaths in China^4^. HCC in China accounts for more than 50% out of incidence around the world^5^. Since HCC are with great invasiveness of HCC^6^, great progresses have been made in more than 70% of patients after the diagnosis of 1 year^7^. Therefore, therapy against advanced HCC is less efficient and survival of patients with advanced HCC is low^8^. Accordingly, it is of great significance to find novel antitumor medicine to prevent the HCC invasion.

Propofol (2, 6-diisopropylphenol) is a commonly used short-term sedative anesthetic^9^. Recently, its potential clinical application other than anesthesia attracts more attentions. Studies have shown that propofol could inhibit the tumor progression^10–13^. In HCC, propofol can inhibit proliferation, migration and invasion of liver cancer cells^14^. Meanwhile, propofol inhibited tumor progression of hepatocellular carcinoma xenografts in BALB/C mice^15^. Propofol also induces apoptosis of hepatocellular carcinoma cells^16^. All these studies indicated that propofol maybe a candidate drug for liver cancer. However, its underlying mechanismof the anticancer effect remains elusive.

As concluded above, we investigated the role of propofol in HCC by regulating the NET1 expression. NET1 silencing decreases the formation of new blood vessels and development of cervical squamous cell carcinoma^17^. NET1 also regulated chemoresistance in bladder cancer cells^18^. Thus, NET1 may correlate with the tumor progression and growth. Here, we indicated the inhibitory effect of propofol on HCC cell invasion and migration mediated by NET1. Meanwhile, NET1 also affects the expression of p-ERK1/2 and VEGF. Thus, this work validated the potential value of propofol in the treatment of liver cancer.

## Methods and materials

### Collection of HCC tissues and maintenance of HCC cell lines

All experimental procedures were approved by the Shanghai Xuhui Hospital Ethics Committee, while human tissue experiments were conducted with the patients’ written consent. All tumor tissues were kindly provided by Shanghai Xuhui hospital. The Ethics Committee of this hospital approved all bioassays and all patients signed the written consent. All experiments were performed in accordance with Shanghai Xuhui Hospital’ guidelines and regulations. And all participants were informed consent for study participation. HEK293 cell line and hepatic cancer cell lines Hep-G2, Huh7, SMMC-7721 and HL-7702 were purchased from ATCC. All HCC cell lines were maintained and passaged in Dulbecco's Modified Eagle's Medium (Gibco) added with 10% fetal bovine serum (FBS; HyClone), and incubated in a warm and moisture refrigerator supplied with 5% CO_2_.

### NET1 silencing by RNAi

The p Silencer™ siRNA expression vector (ThermoFisher) was applied to clone NET1 siRNA or its specific scramble control. The recombinant plasmids were transfected into SMMC-7721 cells using Lipofectamine 2000 (Life Technologies, USA) based on the user guidelines. 24 h later, qRT-PCR and western blot were utilized to measure the silencing effect of siRNA respectively.

### Western blot

Twenty ug of total cell lysates were quantified and separated on polyacrylamide gel, and then transferred to a polyvinylidene difluoride (PVDF) membrane. Then, the PVDF membrane was preincubated with 5% nonfat dry milk prepared by 1 × TBST for 1 h at room temperature, and then incubated with the specific primary antibodies against NET1, p-ERK1/2, ERK1/2, VEGF and GAPDH (purchased from Cell Signaling Technologies, USA) respectively. Then membrane was then incubated with peroxidase-conjugated anti-rabbit or anti-goat IgG (purchased from ThermoFisher). These protein bands were visualized by adding ECL solution droply (Amersham Biosciences).

### RNA extraction and qRT-PCR

TRIzol reagent (ThermoFisher) was used to extract total RNA from HCC cell lines or tissues according to the manufacturer’s user guidelines. Reverse transcription (RT) and one-step RT-PCR kit (Takara) were used to synthesize the first strand of cDNA. qRT-PCR was performed by SYBR Green and ABI apparatus (Thermofisher) according to the principles of manufactures. The relative level of gene expression was calculated by the 2^−∆∆Ct^ method. Primers forward and reverse are chemically synthesized as follows: NET1, 5′-CTG TTC ACC TCG GGA CAT TT-3′ and 5′-TGG AGC TGT CAG ACG TTT TG-3′, GAPDH (5′-GGTATCGTGGAAGGACTCATGAC-3′ and 5′-ATGCCAGTGAGCTTCCCGT TCAGC-3′).

### MTT assay

MTT was used to detect the effects of propofol on SMMC-7721 cell lines. Cells treated with the indicated propofol at doses of 0, 10, 25, 50, 100 μM for 48 h, and then incubated with MTT at room temperature for 4 h to produce formazan. Then, SDS-HCl was used to dissolve the formazan, and the absorbance at 570 nm was measured with a Universal Microplate Reader (Thermo). The cell viability was calculated by the following formula: Value of OD = OD of propofol-treated group/OD of blank control group.

IC50 values were the dose required to propofol inhibitory activity of 50% of the cell population and calculated from logarithmic sigmoidal dose–response curves generated using GraphPad Prism 5.0 software (GraphPad Inc).

### Scratch wound assay

SMMC-7721 cells treated with or without propofol were seeded on 60 mm tissue-culture plastic dishes. When cells reached 80% cell confluence, a scratch wound was created using a sterile pipette tip. At 12, 24 and 48 h post-wound scratching, the cells among different groups were stained with 0.1% Crystal Violet and photographed in the same field of view. Wound closure was calculated according to the ratio of areas uncovered by cells before and after wound scratching.

### Invasion assay

24-well plate of BD transwells was utilized to perform invasion assay. In brief, 5 × 10^4^ cells in 100 μl serum-free medium were seeded on the upper chamber of matrigel-coated transwell, which were embedded into medium of the lower chamber. Six hundred μL 10% FBS serum medium was added to the lower chamber as a chemoattractant. After 48 h incubation, non-migrating cells were removed by wiping the upper chamber with a cotton swab. Migrated cells on the bottom side of the well were stained with Giemsa, and were counted in five random fields under a microscope (Olympus, Japan) at 40 × magnification.

### Statistical analysis

Each assay was repeated at least in triplicates. Data were scientifically analyzed by Microsoft Excel and Graphpad Prism software, and presented as mean ± SEM. The intensity of western blot bands was measured by Image J software. Unpaired t test or one-way ANOVA was used to determine significant differences between two groups or multiple groups respectively.

## Results

### Differential expression of NET1 in hepatocellular carcinoma

**Figure 1:**
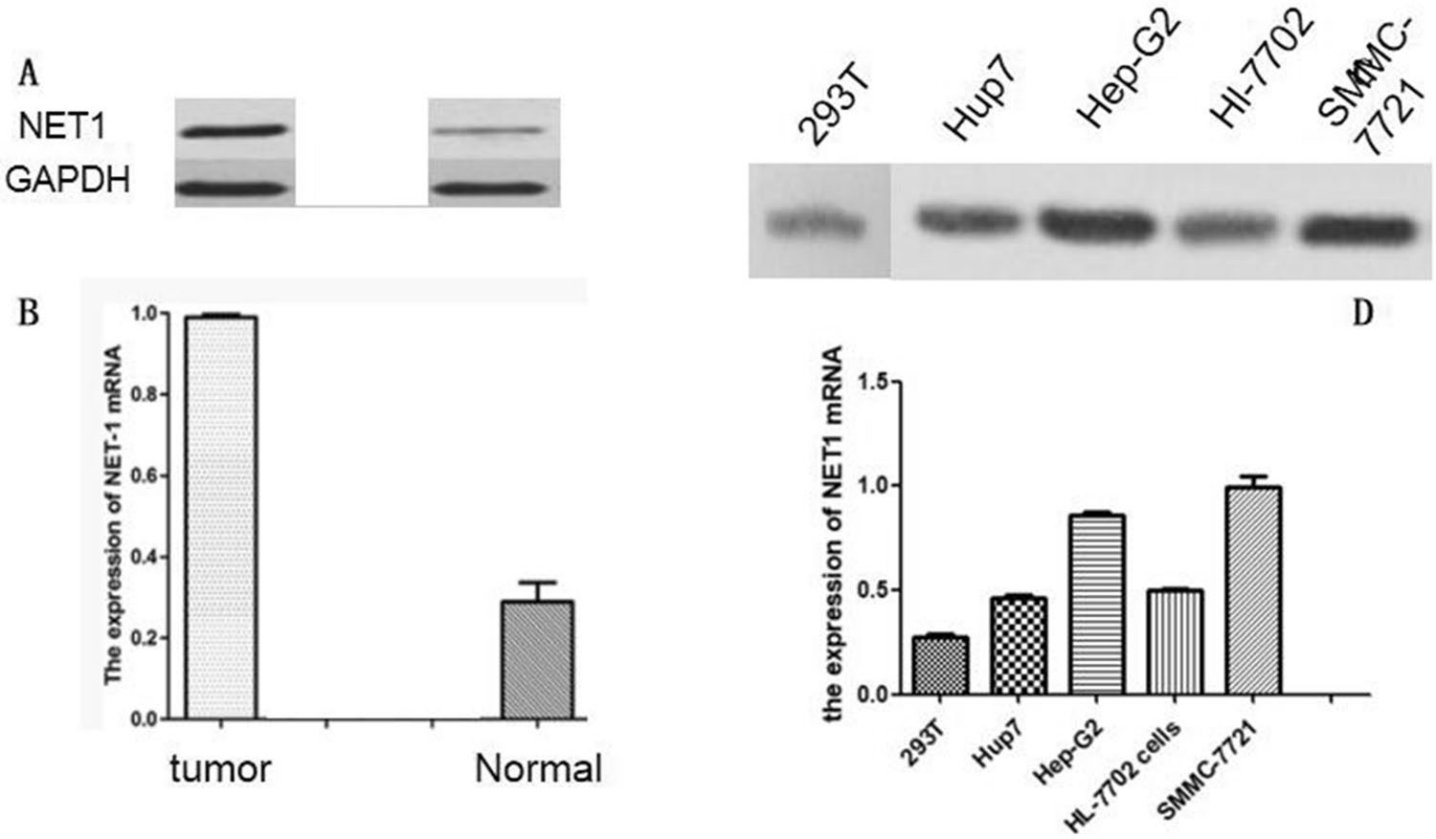
Aberrant expression of NET1 in hepatocellular carcinoma. (**A**, **B**) NET1 was significantly increased in hepatic tumor compared to the normal tissue. (**C**, **D**) NET1 expression was obviously elevated in hepatic cancer cell lines compared to that in HEK293 cell line.

To determine if NET1 was differentially expressed in hepatocellular carcinoma, we measured NET1 expression level in hepatocellular carcinoma tissue as well as hepatic cancer cell lines. The results of western blot analysis further confirmed that the relative expression of NET1 was higher in hepatic tumor when compared with that of normal tissue (Fig. 1A,B). Meanwhile, we also found that NET1 level in hepatic cancer cell line including Hep-G2, Huh7, SMMC-7721 and HL-7702 was significantly higher than that in normal HEK293T cells (Fig. 1C,D). Thus, we concluded that NET1 was significantly higher in hepatic tumors and cancer cell lines, implying its potential role in hepatic tumor growth.

### Propofol suppressed hepatocellular carcinoma growth, migration and invasion

**Figure 2:**
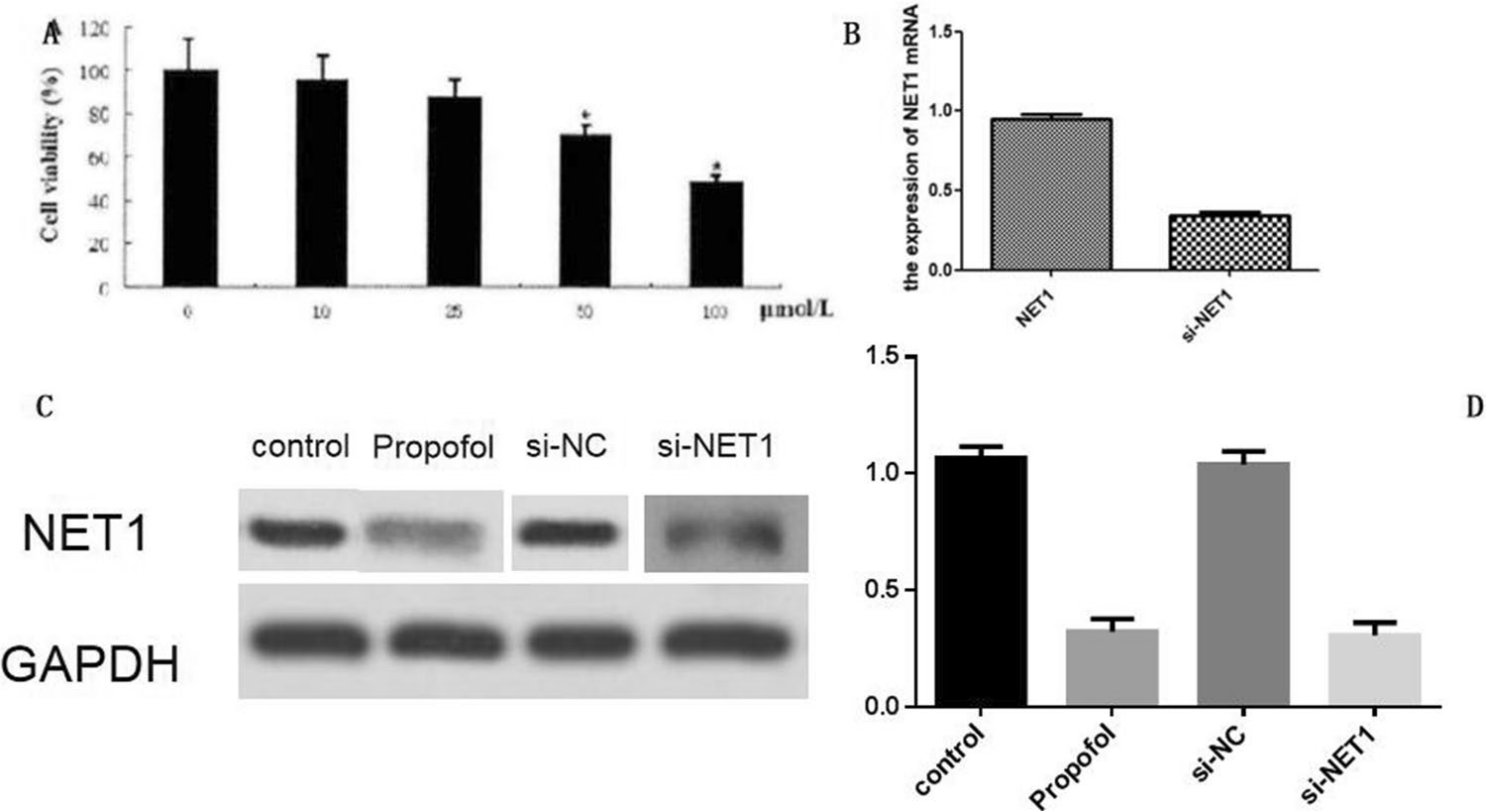
Propofol inhibited SMMC-7721cell viability through regulating NET1 expression. (**A**) Cells treated with the indicated propofol at doses of 0, 10, 25, 50, 100 μM for 48 h exhibited decreased cell viability. (**B**) NET1 mRNA level was decreased by NET1-siRNA. (**C**, **D**) NET1 protein level was decreased by propofol treatment and NET1-siRNA.

**Figure 3:**
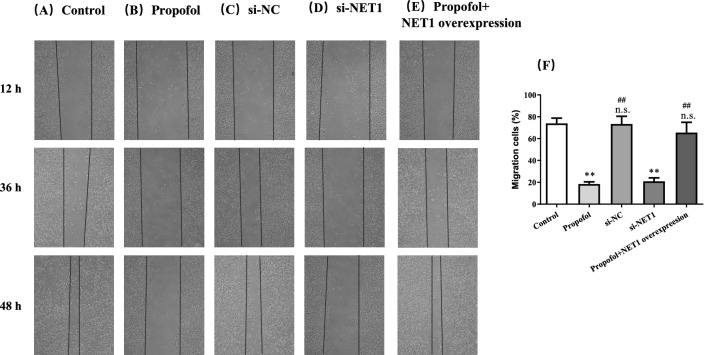
The treatment of 100 μM Propofol (Panel **B**) or NET1 silencing (Panel **D**) significantly inhibited SMMC-7721 cell migration compared to control group (Panel **A**) and NC group (Panel **C**).While the inhibitory effect of propofol was offset by NET1 overexpression (Panel **E**). Statistical analysis on SMMC-7721 cell migration with indicted treatment (Panel **F**). **P < 0.01 compared with control group, n.s. represented no significance compared with control group, # and ## denote P < 0.05, 0.01 compared with si-NET1 group.

Then, we asked whether propofol affected hepatocellular carcinoma growth. Since the NET1 expression in SMM-7721 cells was the highest among HCC cell lines, SMM-7721 cells were employed for the subsequent experiments. The CCK-8 assay showed that SMMC-7721 cell viability was significantly impaired in the presence of 50 or 100 μM propofol (Fig. 2A).The IC50 of propofol was calculated as 91.12 ± 3.27 μM. Considering the cell viability was reduced to almost 50% when cells were incubated with 100 μM propofol, the concentration of propofol in subsequent experiments was set as 100 μM. Furthermore, propofol treatment led to a significant decrease in NET1 level (Fig. 2B–D). This showed that the repressive property of propofol on hepatic tumor growth was highly associated with NET1 expression. In the presence of propofol, migration of SMMC-7721 cells was negatively affected (Fig. 3A,B). By contrast, this inhibitory effect was offset with NET1 overexpression in SMMC-7721 cells (Fig. 3E). However, when NET1 was silenced, the migratory capacity in SMMC-7721 cells was negatively regulated (Fig. 3D) compared to that in scramble control group (Fig. 3C). Meanwhile, the effect of NET1 silencing on SMMC-7721 migration was comparable with that in propofol-treated cells, showing that downregulation of NET1 led to impairment in hepatic cancer cell migration.

**Figure 4:**
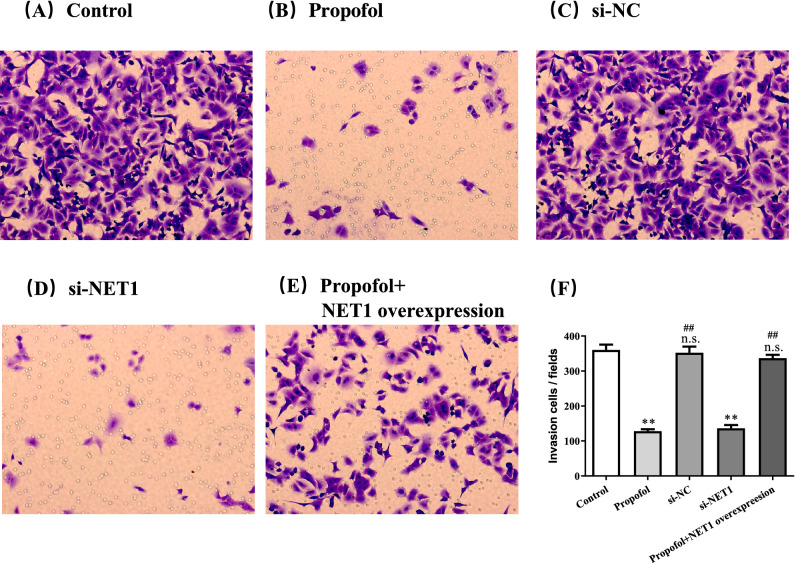
The treatment of 100 μM Propofol for 48 h (Panel **B**) or NET1 silencing (Panel **D**) significantly inhibited SMMC-7721 cell invasion compared to control group (Panel **A**) and NC group (Panel **C**).While (**E**) the inhibitory effect of propofol was offset by NET1 overexpression (Panel **E**). Statistical analysis on SMMC-7721 cell invasion with indicted treatment (Panel **F**). **P < 0.01 compared with control group, n.s. represented no significance compared with control group, # and ## denote P < 0.05, 0.01 compared with si-NET1 group.

Transwell assay indicated that propofol treatment inhibited invasion of SMMC-7721 cells (Fig. 4A,B,F). With overexpression of NET1, alteration in invasion by propofol was counteracted (Fig. 4C,F). Meanwhile, NET1 silencing in SMMC-7721 cells inhibited invasion compared to that in cells transfected with scramble control (Fig. 4D–F). Therefore, we dropped a conclusion that propofol or NET1 silencing could inhibit the growth, migration and invasion, while overexpression of NET1 offset the inhibitory effect of propofol on migration and invasion in SMMC-7721 cells.

### Propofol affected NET1 downstream pathway

**Figure 5:**
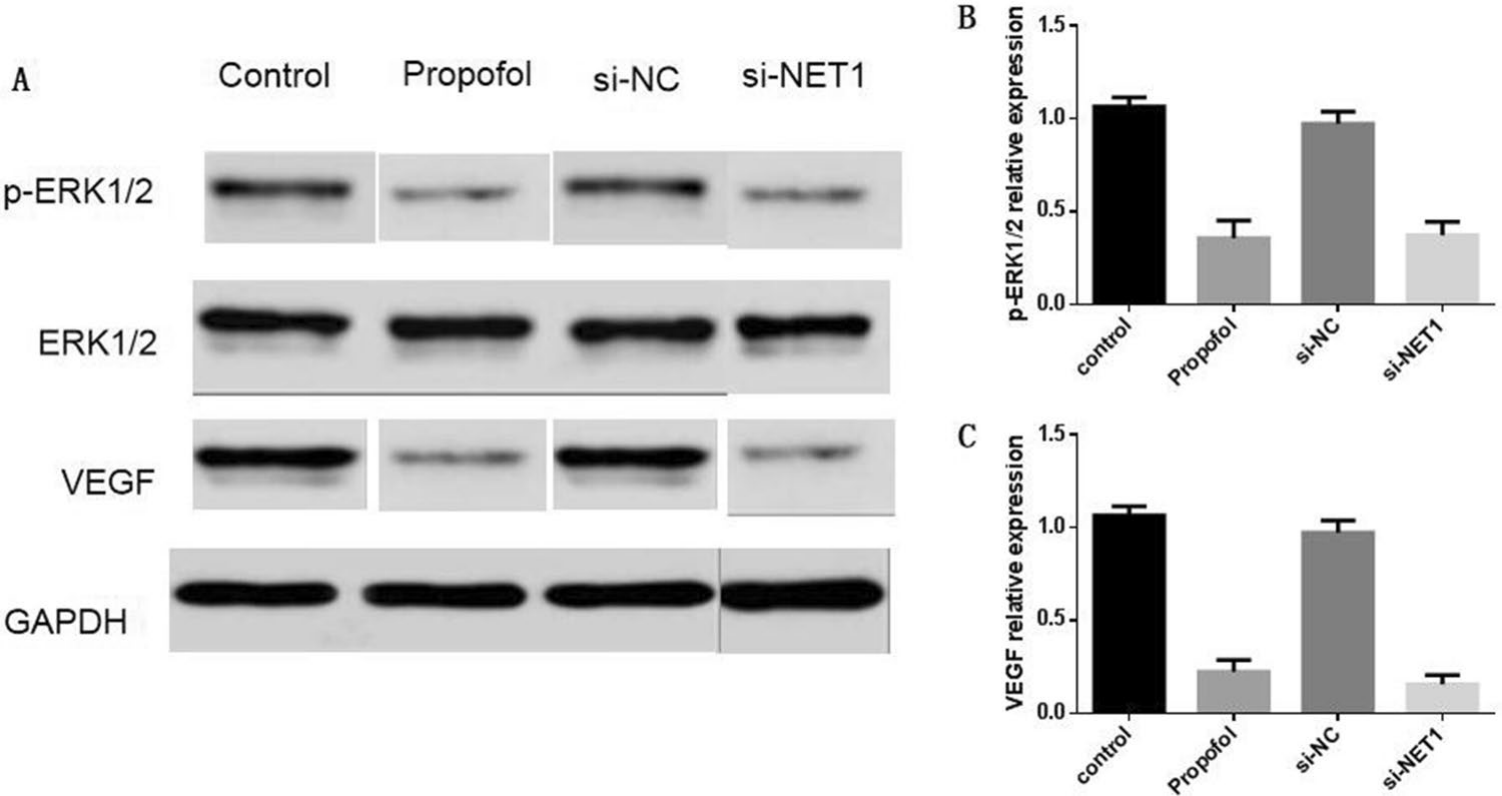
(**A**) The treatment of 100 μM Propofol for 48 h or NET1 silencing downregulated p-ERK1/2 or VEGF protein level. (**B**) Statistical analysis on p-ERK1/2protein level under various conditions. (**C**) Statistical analysis on VEGF protein level under various conditions.

Since propofol treatment regulated NET1 expression, we determined whether it alter the expression level of key elements in NET1 downstream pathway. Western blot analysis showed that propofol decreased phosphorylation level of ERK1/2 in SMMC-7721 cells (Fig. 5). The decrease in phosphorylated ERK1/2 level was also observed when NET1 was silenced (Fig. 5). Meanwhile, propofol or NET1 silencing also decreased VEGF level (Fig. 5). Therefore, propofol treatment affected NET1 downstream target genes.

## Discussion

In this study, we found NET1 regulated propofol-induced inhibitory effect on liver cancer cell invasion and migration. This work provided mechanistic evidence to support this inhibitory effect was mediated by ERK/VEGF signaling. Thus, a new mechanism underlying the influence of propofol on HCC cell migration and invasion was disclosed and this implied potential new therapeutic target against HCC.

A bunch of studies reported therapeutic role of propofol in cancer development. In lung cancer cells, propofol induced apoptosis depending on ERK1/2 signaling^19^. This is consistent with our findings that propofol could inhibit cell viability and decrease the p-ERK1/2 level. It was also reported that silencing of NET1 and VEGF inhibited HCC growth^20^. This was also similarly shown in the current study that NET1 silencing inhibited the invasion and migration in HCC cells. More importantly, we integrated propofol, NET1 and ERK/VEGF and disclosed the correlation among them to regulate the HCC cell viability, migration and invasion.

NET1 is a new member of the tetraspanins group and correlated with malignant tumor development^21^. To elucidate the molecular mechanism underlying NET-1, we examined and found that siNET-1 could regulate the VEGF and p-ERK1/2 level. HCC express a large number of VEGF to initiate angiogenesis to that it receives enough oxygen and nutrients supply^22^. VEGF is a major contributor to the tumor progression and therefore, controlling the VEGF expression may function as a promising mean to treat the HCC. Since propofol treatment or siNET1 decreased the VEGF level, further indicating propofol may function as a potential anti-HCC medicine and NET1 is likely to become a therapeutic target to HCC. Thus, more works will be focused on the in vivo study to show whether propofol or NET-1 will regulate the HCC growth and invasion in xenografted tumors.

### Ethics, consent and permissions

All experimental procedures were approved by the Shanghai Xuhui Hospital Ethics Committee, while human tissue experiments were conducted with the patients’ written consent.

### Consent to publish

All authors agreed to publish the study.
